# GPU acceleration of Darwin read overlapper for de novo assembly of long DNA reads

**DOI:** 10.1186/s12859-020-03685-1

**Published:** 2020-09-17

**Authors:** Nauman Ahmed, Tong Dong Qiu, Koen Bertels, Zaid Al-Ars

**Affiliations:** 1 Delft University of Technology, Delft, Netherlands; 2 University of Engineering and Technology Lahore, Lahore, Pakistan

**Keywords:** Genomics, Read overlapper, De novo assembly, Long DNA reads, GPU acceleration

## Abstract

**Background:**

In Overlap-Layout-Consensus (OLC) based de novo assembly, all reads must be compared with every other read to find overlaps. This makes the process rather slow and limits the practicality of using de novo assembly methods at a large scale in the field. Darwin is a fast and accurate read overlapper that can be used for de novo assembly of state-of-the-art third generation long DNA reads. Darwin is designed to be hardware-friendly and can be accelerated on specialized computer system hardware to achieve higher performance.

**Results:**

This work accelerates Darwin on GPUs. Using real Pacbio data, our GPU implementation on Tesla K40 has shown a speedup of 109x vs 8 CPU threads of an Intel Xeon machine and 24x vs 64 threads of IBM Power8 machine. The GPU implementation supports both linear and affine gap, scoring model. The results show that the GPU implementation can achieve the same high speedup for different scoring schemes.

**Conclusions:**

The GPU implementation proposed in this work shows significant improvement in performance compared to the CPU version, thereby making it accessible for utilization as a practical read overlapper in a DNA assembly pipeline. Furthermore, our GPU acceleration can also be used for performing fast Smith-Waterman alignment between long DNA reads. GPU hardware has become commonly available in the field today, making the proposed acceleration accessible to a larger public. The implementation is available at https://github.com/Tongdongq/darwin-gpu.

## Background

DNA sequencing techniques used today produce short pieces of data (called reads) that represent parts of the sampled DNA, possibly containing some errors. The length and error rate of these reads depends on the sequencing technique used. DNA assembly tries to combine the reads into larger, more accurate DNA segments. For these DNA reads, graph-based assemblers are used for the assembly process, which comes in two flavors: Overlap-Layout-Consensus (OLC) and de Bruijn Graph (dBG).

The OLC assemblers [1] first find overlaps and build an overlap graph. Each node represents a read, and each edge represents an overlap between two reads. During the layout phase, the graph is analyzed to find paths, corresponding to segments of the original genome. The perfect graph contains one path that visits each node exactly once. This problem can be described as finding a Hamiltonian Path. A Hamiltonian Path includes all vertices of a graph exactly once. Examples of assemblers that use the OLC approach are Dazzler [2] and SGA [3].

In dBG based assemblers [4], each read is divided into *K*-*mers*. A *K*-*mer* is a substring of a read having a length *K*. Each *K*-*mer* represents a directed edge between two vertices, where the source vertex represents the first *K*−1 bases of the *K*-*mer*, and the destination vertex the last *K*−1 bases of the *K*-*mer*. When a particular *K*−1 vertex does not exist, it is created, otherwise, the existing one is reused. The weights of the edges indicate how many times a particular *K*-*mer* is encountered. The next step is to find an Eulerian Path, which is a path that includes all edges of a graph exactly once. Examples of dBG assemblers are Velvet [5], ABySS [6] and SOAPdenovo2 [7].

So called Next Generation Sequencing (NGS) techniques produce reads with lengths anywhere from 50 to 500 base pairs. They can be produced at high throughput but at the expense of a smaller read length. However, DNA can contain repeat regions, where a certain piece of DNA is repeated many times back-to-back, or a repeat could appear in many different places in the genome. Since these repeats can be longer than the produced short reads, this means the reads cannot be used to resolve these repeat regions. Third generation sequencing produces much longer reads, of up to 60K base pairs. Due to their length, these reads are more likely to contain a whole repeat region, which makes them suitable for accurately reconstructing the repeat regions. A major drawback of longer DNA reads is their higher error rate, ranging from 15-30%, depending on the exact sequencing technology. dBG based assembly is the more preferred approach for NGS reads, which are much shorter and have much lower error rates. However, de Bruijn Graphs are quite susceptible to sequencing errors, since one substituted base pair causes *K* incorrect *K*-*mers*. Pair this with an often-used values of *K* above 50 and third generation sequencing error rate of about 15%, it is clear that the graph will contain a lot of incorrect edges. Therefore, OLC based assemblers are more suitable for state-of-the-art third generation long DNA reads produced by Pacbio and Oxford Nanopore sequencers.

Darwin [8] is a read overlapper for the assembly of long DNA reads. Darwin is designed to be highly accurate, achieving a sensitivity of 99.89% and a precision of 88.30% for simulated Pacbio reads. This is higher than other commonly used read overlappers such as Daligner [2]. The ASIC (Application-Specific Integrated Circuit) implementation of Darwin is shown to be hundreds of times faster than other software based overlappers. However, ASIC implementation requires bulk volume production to be economically feasible. Moreover, DNA analysis using high-throughput DNA sequencing is an evolving field, and any major improvement in the algorithm will require a new ASIC implementation which costs both time and money.

Heterogeneous systems with GPU accelerators have become easily accessible due to their widespread use. They have shown convincing speedups in many high performance computing applications. Therefore, GPU acceleration of various genome analysis algorithms has been the topic of many research works, like in [9, 10] and [11]. In this paper, we present a GPU accelerated version of Darwin. We identified the computational bottleneck in the Darwin software and replaced it with the GPU accelerated version. The accelerated implementation proposed in this paper is orders of magnitude faster than its software counterpart. The contributions of the paper are as follows:

- The paper shows the GPU implementation of the Darwin read overlapper used in the de novo assembly of long DNA reads.
- The paper shows that the GPU acceleration of Darwin is orders of magnitude faster than the multithreaded software version on both IBM Power8 and Intel Xeon machines using a real Pacbio dataset.
- The results in the paper show that the GPU implementation of Darwin can also be applied for accelerating Smith-Waterman alignment of long DNA reads.

## Background

Smith-Waterman (SW) [12] algorithm finds local alignment between a pair of sequences. Smith-Waterman is exact, producing the optimal local alignment. It can be implemented using dynamic programming which computes a 2D matrix *S*. Let *V* and *W* be the two sequences to be aligned. Let $V_{0}, V1, \dots, V_{|V|-1}$ and $W_{0}, W1, \dots, W_{|W|-1}$ be the bases of *V* and *W*, respectively. |*V*| and |*W*| are the lengths of *V* and *W*. *S*(*i*,−1)=*S*(−1,*j*)=0 for $i=0, 1, 2 \dots, |V|-1$ and $j=0, 1, 2 \dots, |W|-1$. The cells in the matrix are computed using the following recurrence relation:

1 $$\begin{array}{*{20}l} S(i,j) =& max\left\{\begin{array}{lccc} S(i-1,j) + gap \\ S(i,j-1) + gap \\ S(i-1,j-1) + subt\left(V_{i},W_{j}\right) \\ 0 \\ \end{array}\right. ~ \end{array} $$

2 $$\begin{array}{*{20}l} m_{i,j} =&\left\{\begin{array}{cc} (i, j) & S(i,j) > m \\ m_{i,j} & S(i,j) \le m \\ \end{array}\right. ~ \end{array} $$

3 $$\begin{array}{*{20}l} m =& max\left\{\begin{array}{cc} m \\ S(i,j) \end{array}\right. ~ \end{array} $$

4 $$\begin{array}{*{20}l} D(i,j) =&\left\{\begin{array}{clcc} 0 & S(i, j) = 0 \\ \uparrow & S(i, j) = S(i-1, j) \\ \leftarrow & S(i, j) = S(i, j-1) \\ \nwarrow & S(i, j) = S(i-1,j-1) + subt\left(V_{i},W_{j}\right) \\ \end{array}\right. \end{array} $$

Here, *S* and *D* are the score and traceback matrices, respectively. *match*, *mismatch* and *gap* are numeric parameters. *subt*(*V*_*i*_,*W*_*j*_) is equal to *match* if *V*_*i*_=*W*_*j*_, and is equal to *mismatch* otherwise. *gap* is the penalty for inserting a gap. *m* is the alignment score, which is initialized to zero, and *m*_*i*,*j*_ is the corresponding position on *V* and *W*. The traceback matrix is required to compute the actual alignment. Traceback starts from the highest scoring cell and follows the arrows in *D* until a zero or a boundary of the matrix is encountered. Equations 1 and 3 indicate that for computing the alignment score *m* there is no need to store the whole *S* matrix as all cells of the *S* matrix are computed using only the values of three other cells *S*(*i*−1,*j*), *S*(*i*,*j*−1) and *S*(*i*−1,*j*−1). Hence, to compute the alignment score, storing only the values in the previous row and column are sufficient to compute *m*. The above equations are for calculating the alignment with a linear-gap scoring model. However, Darwin and our GPU implementation also support the more commonly used affine gap penalty model in which there are separate penalties for opening a gap (*gapo*) and extending a gap (*gape*).

A straightforward way of finding all overlaps is performing an alignment algorithm, such as Smith-Waterman, on every pair of reads. The number of alignments is quadratic with the number of reads, and the runtime of one alignment is quadratic with the lengths of the involved reads, making this method not feasible. Many heuristic algorithms have been developed to perform this alignment, for different lengths and error rates. Seed-and-extend is one heuristic, which dramatically reduces the amount of computation needed [13]. A seed is a *K*-*mer* made up of *K* consecutive bases of a read. Instead of performing Smith-Waterman on each read pair, only read pairs that have one or more common *K*-*mers* are aligned. A common *K*-*mer* between two or more reads is known as a “seed hit”. Darwin also uses the seed-and-extend approach, which reduces the amount of computation needed, without compromising the output by much. Other algorithms, like BLAST [14], also use the seed-and-extend approach, but give sub-optimal alignments. Results in [8] show that Darwin provides optimal Smith-Waterman alignments between long DNA sequences with error rates up to 40%.

### Darwin

**Fig. 1:**
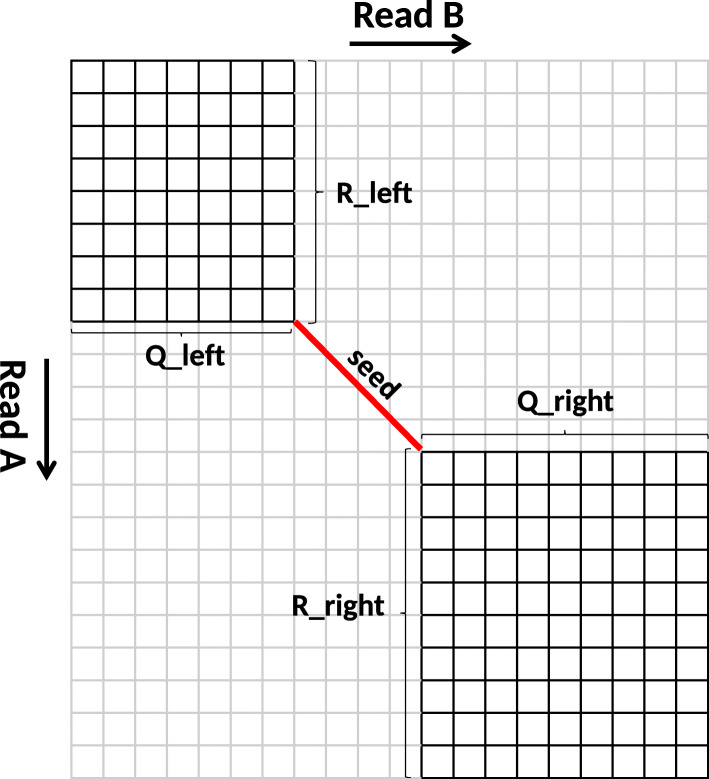
The seed-and-extend method used in Darwin to find the overlap between *Read A* and *Read B*. *R_left* and *R_right* are the substrings of Read A. *Q_left* and *Q_right* are the substrings of Read B

Darwin is read overlapping algorithm for de novo assembly of third-generation long DNA reads. It is based on the seed-and-extend. It consists of a filter called D-SOFT (Diagonal-band Seed Overlapping based Filtration Technique), which finds seed hits, and GACT (Genome Alignment using Constant memory Traceback), which extends the seed hit by performing sequence alignment between the sequences on the left and right of the seed hit. Figure 1 shows the seed-and-extend technique employed in Darwin to find the overlap between *Read A* and *Read B*. To compute the overlap, a seed hit is extended on both sides by aligning *R_left* with *Q_left* and *R_right* with *Q_right*. This speedups the computation by avoiding the computation of a large number of dynamic programming matrix cells (grey cells in Fig. 1).The dynamic programming matrix computed to align *R_left* with *Q_left* is known as *left extension matrix*. Similarly, the dynamic programming matrix computed to align *R_right* with *Q_right* is known as *right extension matrix*.

#### D-SOFT

D-SOFT is the seeding stage of Darwin, also known as the *filtering* stage. Darwin uses minimizers [15] as seeds which are *K*-*mers* extracted from all the reads to be overlapped. The position of a seed is stored in a minimizer table which records the location of the seed in a read along with the identifier of the read. The window size *w* is the most important parameter for building a minimizer table and must be smaller than the seed length (*K*). To obtain seed hits, ‘*N*’ *K*-*mers* of a read are used as seeds that are located in other reads using the minimizer table. Two reads are considered for alignment in the extension phase if they have at least *h* unique bases in common. The pair of reads passing this filter are aligned using a modified Smith-Waterman algorithm described below.

#### GACT

The seed-and-extend approach to find overlap between two reads is much faster than performing the complete Smith-Waterman alignment algorithm. However, the reduced dynamic programming matrices could still be quite large. State-of-the-art third generation sequencers produce reads having lengths in megabases and the dynamic programming matrix in the seed extension may have around 1 Tera cells to compute. For example, if the seed hit lies at the beginning of the two reads, i.e. near the top left corner in Fig. 1, the right extension matrix is nearly as large as the full matrix.

**Fig. 2:**
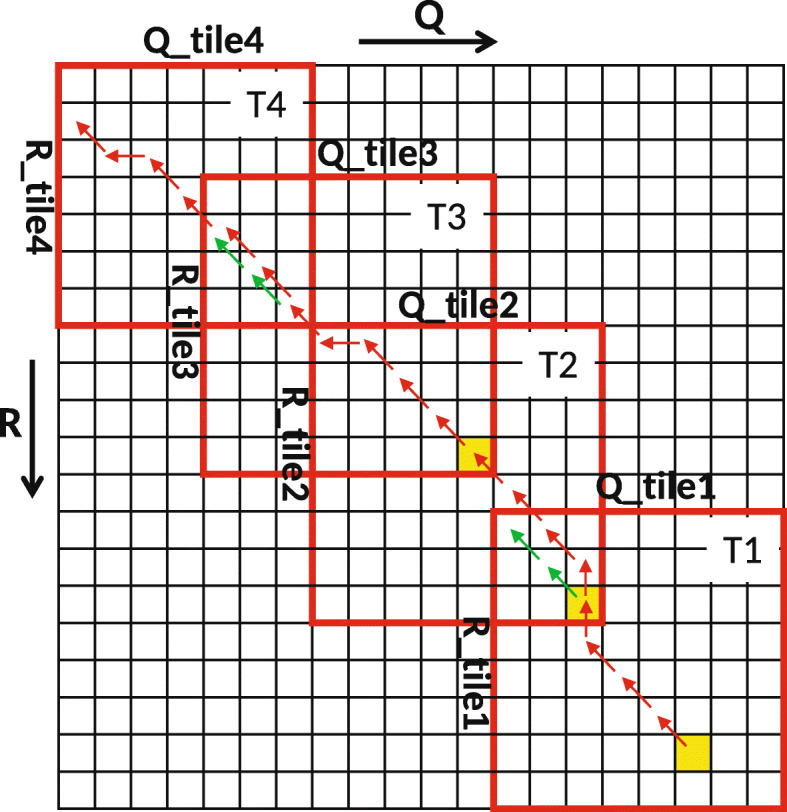
An example of the GACT algorithm


Numerous efforts to accelerate Smith-Waterman have been made, both by using hardware like [16] and software [17]. But the memory required to store the traceback matrix *D* is still an issue. One can apply the Hirschberg’s algorithm described in [18] to reduce the RAM storage but at the cost of increase in computation time. Therefore, Darwin proposed the GACT algorithm for seed extension. It has two advantages: 1) All the cells of the right and left extension matrix are not computed reducing the computation time. 2) The traceback matrix is very small. GACT performs normal Smith-Waterman on a submatrix of the extension matrix, known as *tiles* of size *T*x*T*. After computing a tile it computes the next tile, which overlaps the previous tile with at least *O* cells on both axes. For reasonable values for *T* and *O*, GACT has shown to produce the same result as normal Smith-Waterman [8]. Figure 2 shows an example of computing the extension matrix with the GACT algorithm. In the example *T*=8 and *O*=3. The tiles are computed in the order *T*1,*T*2,*T*3 and *T*4. The example in Fig. 2 can be used to explain both the computation of left and right extension matrices. The only difference is $R = \overline {R\_right}$ and $Q = \overline {Q\_right}$ in case of right extension, where $\overline {R\_right}$ and $\overline {Q\_right}$ is the reverse of *R_right* and *Q_right* sequences, respectively. Listing 1, shows the algorithm for left extension. Positions *i*_*curr* and *j*_*curr* are produced by D-SOFT. The start and end position of the current tile are stored in (*i*_*start*,*j*_*start*) and (*i*_*curr*,*j*_*curr*), respectively. The traceback path of the whole left extension is kept in *tb_left*. The function *Align*() uses Smith-Waterman to compute traceback matrix *D* between subsequences *R_tile* and *Q_tile*. Once the traceback matrix is filled, traceback is performed starting from the bottom-right cell, except for the first tile, where traceback starts from the highest-scoring cell. The starting cells of the traceback are coloured yellow in Fig. 2. *Align*() returns the number of bases in *R* and *Q* aligned by this tile (*i_off*,*j_off*), the traceback arrows/pointers (*tb*) and the position of the highest-scoring cell (*i_max*,*j_max*). *Align*() also limits *i_off* and *j_off* to at most *T*−*O* bases, to ensure the next tile overlaps by at least *O* bases on both *R* and *Q*. The green arrows shows the path taken by the traceback in a tile if there is no limit and the traceback is allowed to complete. The left extension finishes when it hits the end of *R* or *Q*, or when traceback cannot add any bases to the existing alignment. The memory needed for the traceback is $\mathcal {O}\left (T^{2}\right)$, which is constant since *T* is chosen upfront. The whole alignment of the extension is contained in *tb_left* and is equivalent to the path traced by the red arrows in Fig. 2. The alignment score of the extension can also be computed with the help of *tb_left* The right extension operates on the reverse of *R* and *Q*.

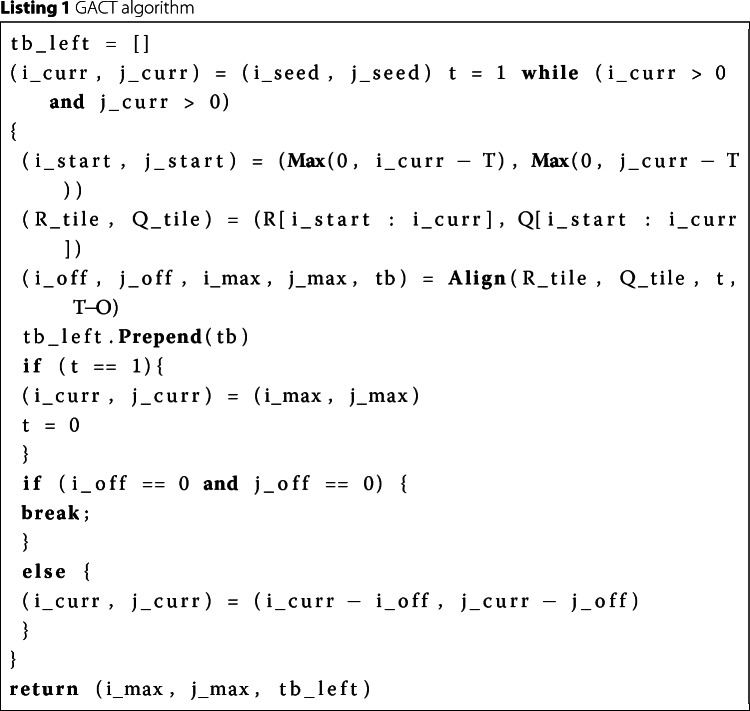


The performance of GACT is linear (*O*(*max*{|*ReadA*|,|*ReadB*|}·*T*)) where |*ReadA*| and |*ReadB*| are the lengths of *Read A* and *Read B*, respectively. It is more suited for long reads than banded alignment [19] because banded alignment uses a static band around the main diagonal. GACT allows for flexible bands since the position of the new tile depends on the traceback path, this is useful for long reads that have high indel rates.

### GPU processing

A GPU is a Graphics Processing Unit, which is a processor that is mainly used to perform video processing. GPUs contain many cores that allow them to perform parallelizable tasks very quickly. A GPGPU, or General Purpose GPU, can be programmed to perform tasks that are different from video processing. GPUs cannot operate on their own, they must be guided by a CPU. The functions that run on a GPU are called *kernels* and are usually launched by a CPU.

CUDA is a parallel programming platform that allows people to use Nvidia GPUs for their applications. Developers can write kernels, launched from a CPU function. The GPU is referred to as *device* and the CPU as *host*. Kernels can be launched from the CPU with a certain number of *thread blocks* with each block containing many GPU threads. The number of blocks and the number of threads in a block are the kernel launch parameters. Each thread executes the kernel code, although they usually operate on different data.

On a hardware level, an NVIDIA GPU is divided into Streaming Multiprocessors (SM). Each SM contains several cores, or Streaming Processors (SP), these are the basic building blocks and perform the actual calculations. Each block is assigned to at most one SM. This block’s threads are then executed as warps, with 32 threads per warp. Each SM has multiple warp schedulers, so multiple warps can run in parallel on an SM. All threads in a warp must execute the same instruction, if a thread is the only to take a branch, the other threads must wait until the branch is completed, this is called *thread divergence*.

GPUs have several different memory types and levels. It has its own DRAM known as the *global memory* and a cache shared by all SM’s. Accesses to the global memory are also executed in parallel, this means that all threads try to read/write to the memory in parallel. If the addresses are next to each other, only one memory transaction is needed, since a transaction processes a whole memory line. This is known as coalescing. Non-coalesced memory accesses cause multiple memory transactions.

A general workflow using a GPU is:

1. Data is copied from the main memory to the GPU global memory.
2. The CPU launches the GPU kernel.
3. The GPU executes the kernel.
4. Results are copied from the GPU to the CPU memory.

### Previous research

Multiple efforts to accelerate the DNA alignment algorithm on GPU have been made. MUMmerGPU [20] is one of the first GPU accelerated algorithms, it stores a suffix tree of the reference sequence on the GPU, and aligns it with queries. Its newest GPU implementation shows a 13x speedup over the CPU implementation. CUDAlign [21] accelerates the exact Smith-Waterman algorithm and allows an affine gap. The input sequence length is only restricted by the available global memory. It uses linear space and boasts a 702x and 19.5x speedup compared to 1 core and 64 cores, respectively. CUSHAW2-GPU [22] is an accelerated short read aligner. Other work has been done on accelerating BWA-MEM [23] and Protein database search [24].

## Implementation

### Profiling

We measured the runtime of various elements of the Darwin algorithm on the CPU. Two notable parts are D-SOFT (which consists of building the minimizer table, and finding the seeds) and aligning using GACT. Of those two, the *Align*() function in GACT (Listing 1) takes the most time, namely 99.9% for Pacbio reads. Therefore, we accelerated the *Align*() function on GPU. We selected a tile size *T* of 320 as it gives optimal Smith-Waterman alignment scores [8]. With this setting, 63% of the tiles are exactly 320x320. The remaining tiles are smaller as they occur near the edges of *R*-*Q* matrix (Fig. 2). If some GPU threads have a smaller tile size, it will cause some divergence, because they will have to wait until the threads with larger tile sizes are finished.

### Acceleration

**Fig. 3:**
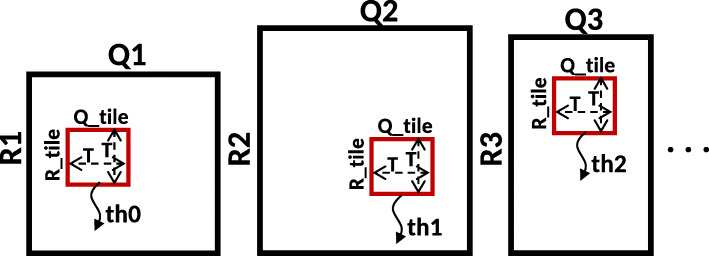
GPU thread assignment for parallelization of the tile computation in GACT. *th* is a GPU thread

**Fig. 4:**
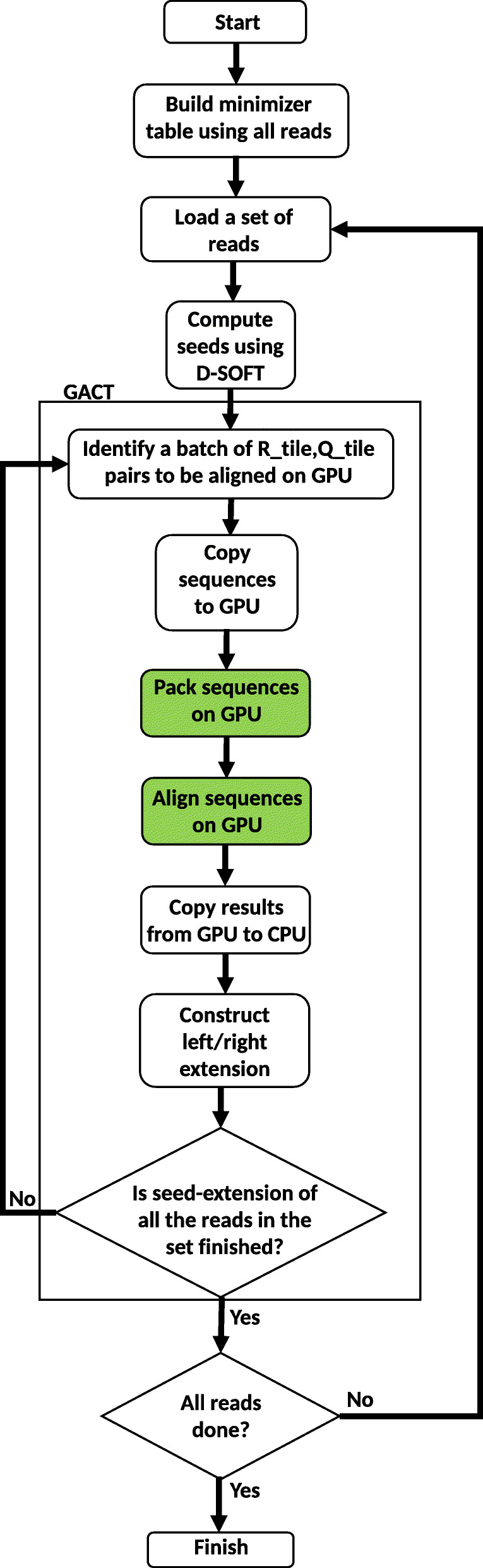
The flow graph of GPU accelerated Darwin. The green boxes are GPU kernels

It is possible to run the whole GACT kernel on the GPU, for both left and right extension. However, since it is not known how long the resulting alignment will be, and all GPU threads have to wait until all threads are done, this will cause lots of idle time. Instead, it is chosen to only have a single tile of size *T*x*T* aligned per GPU-thread per GPU-invocation as shown in Fig. 3. The *Align*() function for many different *R*,*Q* pairs are executed in parallel on the GPU. Figure 4 shows the flow graph of GPU accelerated Darwin. It has two GPU kernels shown by green processes in the flow graph. All other tasks are performed on the CPU. The CPU builds the minimizer table using all the reads for which the overlaps have to be computed. The accelerated algorithm processes a set of reads to exploit the massive parallelism of the GPU. The CPU first computes the seed hits for all the reads in the set using the D-SOFT algorithm. With the help of the seed hit location the sequences for the left and right extension matrices are determined. i.e. (*R_left*,*Q_left*) and (*R_right*,*Q_right*). One tile (*R_tile*, *Q_tile* pair) from each extension matrix is assigned to a GPU thread for alignment. All the tile alignments are computed in parallel on the GPU. There are enough seed hits, and hence sufficient extension matrices in the set of reads to fully utilize all the GPU resources. In the post-processing step, full alignment of the extension using *tb_left* (and *tb_right* for right extension) is constructed on the CPU. As described in the “Profiling” section all the tasks other than computing the alignment between *R_tile* and *Q_tile* takes a negligible amount of time on the CPU.

**Fig. 5:**
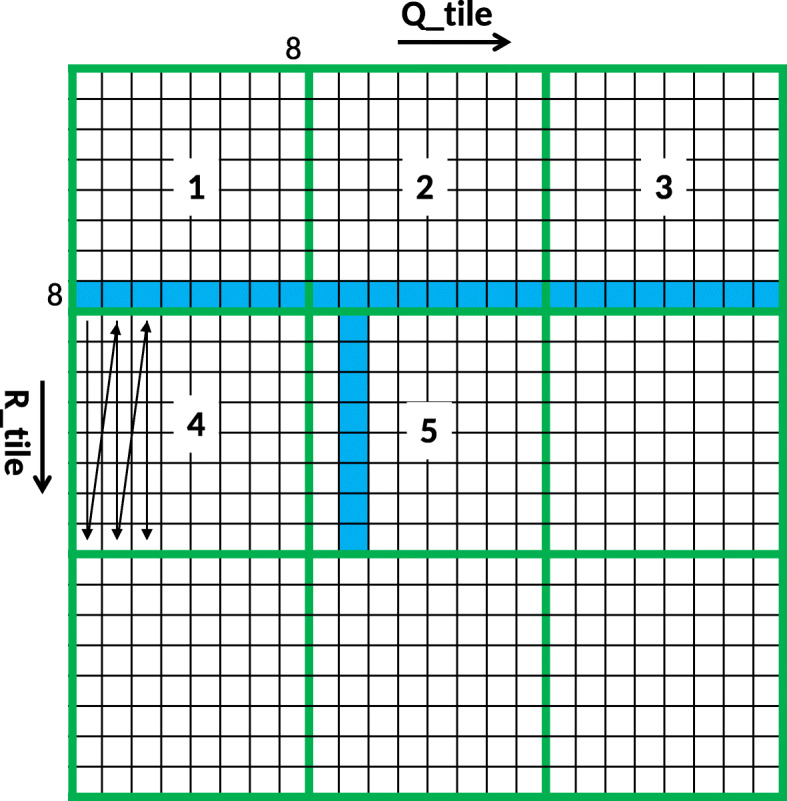
Layout of the tile computed on the GPU. Each green box is a 8x8 submatrix


To reduce the GPU memory accesses, the alignment is preceded by a packing step as indicated in the flow graph of Fig. 4. The bases of both sequences are packed in a 4-bit format, where 8 bases are packed into a 32-bit integer. This packing is performed on the GPU and it is hundreds of times faster than packing bases on CPU [25]. To align *R_tile* with *Q_tile*, we extended the local alignment kernel of GASAL (GPU Accelerated Sequence Alignment Library) library [25]. The tile is subdivided into submatrices of size 8x8. Since there are 8 bases in one integer, only two global memory accesses are required to compute a single submatrix. The layout of a tile computed on the GPU is shown in Fig. 5. Each green box contains an 8x8 submatrix. The submatrices are computed in the order shown by their number. The arrows in the submatrix number 4 show the order of computation of the dynamic programming cells in a submatrix. The figure shows that the *Q_tile* sequence is read multiple times. Hence, packing the sequence with 4 bits per base helps to keep it in the cache for faster access. It is clear from Eqs. 1-4 that to compute a column of the submatrix only the cells in the left column and the row above it are required. The required column and row are colored blue in Fig. 5. The column has only 8 elements, and hence can be stored in GPU registers. Therefore, the total amount of memory required is $\mathcal {O} \left (T + T^{2}\right)$, where $\mathcal {O}(T)$ is required for computing maximum alignment score *m* and $\mathcal {O}\left (T^{2}\right)$ for storing the traceback matrix *D*. Algorithm 1 shows the GPU implementation of the *Align*() function. The pseudocode above Line 4 is for computing the position of maximum alignment score (*i*_*m*_*ax*,*jmax*) and the traceback matrix *D*. Observe that the all the writes in *D* are coalesced to optimize the memory bandwidth and reduce the number of memory transactions. The pesudocode below Line 4 is for computing the traceback path *tb*. The GPU accelerated Darwin supports both linear as well as affine gap penalties. Algorithm 1 shows the alignment using the linear gap penalties. The algorithm with affine gap penalties has a similar layout and omitted here for brevity.

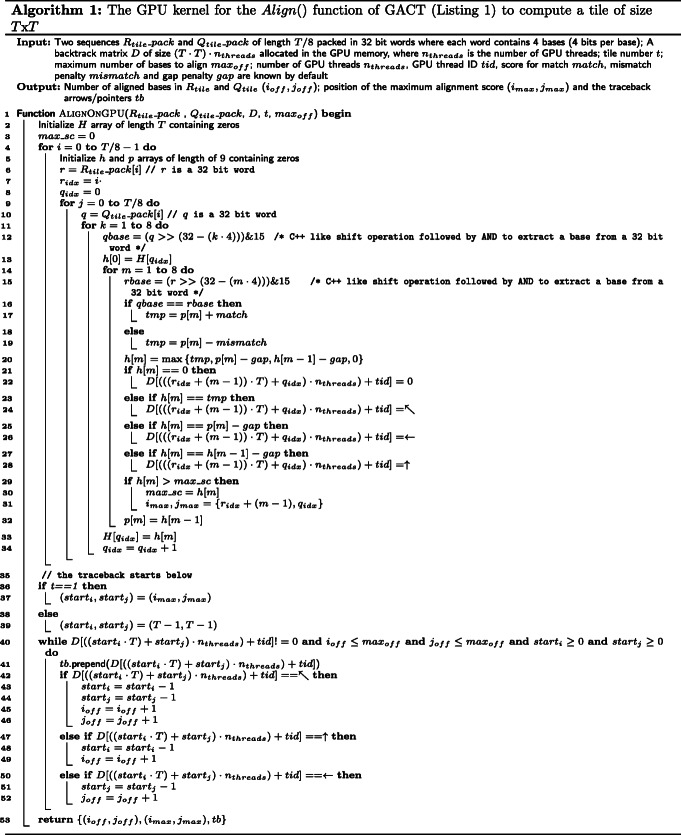


Sequence alignment of long DNA sequence is a performance bottleneck in genome analysis algorithms. The results of Darwin alignment are same as for normal Smith-Waterman for reasonable values of *T* and *O*. Hence, the Darwin algorithm can also be applied for Smith-Waterman alignment between two long DNA sequences and our GPU implementation of Darwin can be used to accelerate Smith-Waterman alignment (with traceback) for long DNA sequences.

## Results

We compared our GPU acceleration with the hand-optimized CPU version of Darwin [26] (commit: 16bdb81). Tests are performed on both IBM as well as Intel machines. The IBM machine (S824L) has 2 sockets with each socket containing a 10-core POWER8 @ 3.42 GHz processor. Each core has 8-way Simultaneous Multithreading. Hence, there are 160 logical cores in total. The machine has 256 GB of RAM and a Tesla K40m. The CUDA version is 7.5, and the operating system is Ubuntu 3.19.0-28-generic. The GCC version is 4.9.2.

The Intel machine has 2 sockets with each socket containing a 6-core Xeon E5-2620 @ 2.4 GHz processor. Each core has 2-way Hyperthreading. Hence, there are 24 logical cores in total. The machine has 32 GB of RAM and a Tesla K40c. The CUDA version is 9.2, and the operating system is CentOS 7.5. The GCC version is 4.8.5. The K40c and K40m have the same performance, the only difference lies in their cooling method.

We use Pacbio 54x Human sequencing data [27]. The data has a size of 172 gigabytes containing 21,856,161 reads. Since the runtime of the experiments has a quadratic relationship with the number of reads, we use first 50 megabytes (8566 reads) as the input dataset to finish the experiments in a reasonable time. Even with 50 megabytes input dataset, the CPU implementation takes more than 2 hours to run on 8 threads of the Intel machine (Fig. 8). The input dataset contains reads up to 33 kilo bases long with an average read length of 6 kilo bases. Darwin computes the overlaps between the reads in the input dataset. The settings for GPU and CPU implementation are as follows: *match*,*mismatch*,*gapo*,*gape*=(1,−1,−1,−1), *N*=800, *T*=320, *O*=120, *K*=14, *h*=21, *w*=1.

**Fig. 6:**
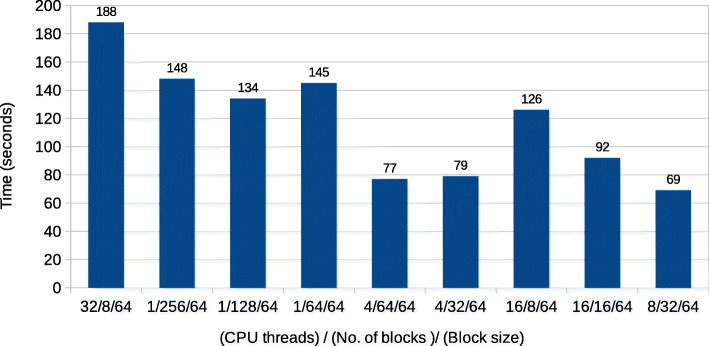
Total execution time of GPU accelerated Darwin for different values of number of CPU threads, numbers of blocks and block size on an IBM machine

Since our GPU implementation accelerates only the *Align*() function on the GPU, everything else is executed on the CPU with multiple threads. Each CPU thread launches a batch of *R_tile* and *Q_tile* sequences to be aligned on the GPU. Since all these CPU threads share a single GPU, it is necessary to investigate how the choice of numbers of CPU threads, number of GPU blocks and the number of threads in a block affect the performance. Figure 6 shows the total execution size for various settings of these factors. The figure shows that the fastest execution time is obtained with 8 CPU threads running with the GPU launch parameters of 32 blocks and 64 threads per block. We performed a similar analysis for the Intel machine and found that 8/32/64 is the best setting in the case of the Intel machine as well. Therefore, we used the 8/32/64 ((Number of CPU threads) / (number of blocks) / (number of threads per block)) setting for running the GPU implementation in the remainder of the experimental results.

**Fig. 7:**
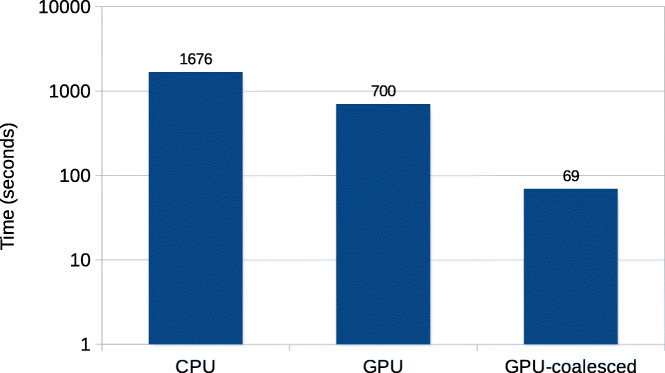
Total execution time of the Darwin’s CPU implementation and GPU accelerated Darwin, on the IBM machine. The CPU implementation is running with 64 CPU threads

Figure 7 shows the total execution time of the CPU implementation of Darwin and compares it with the total execution time of the GPU accelerated Darwin, for the IBM machine. Note that the *y*-axis of the figure represents a logarithmic scale due to the high speedup achieved by the GPU implementation. The CPU implementation is running with 64 threads, which gives nearly the fastest execution time. Two GPU times are reported: “GPU” and “GPU-coalesced”. “GPU” is the time without coalescing the accesses to the traceback matrix *D*. The figure shows that the GPU acceleration without coalescing is 2.4x faster than the CPU implementation. Coalescing further accelerates the GPU implementation by 10x to achieve an overall speedup of 24x.

**Fig. 8:**
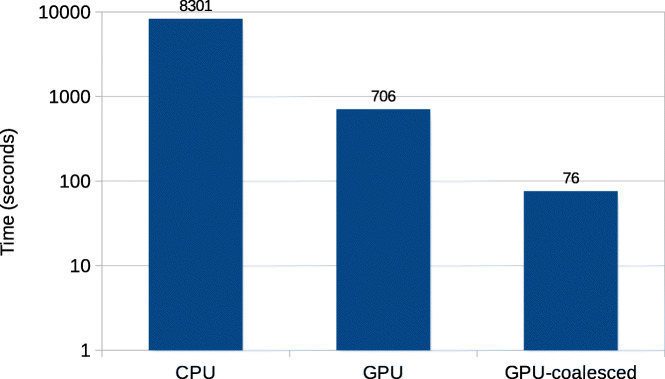
Total execution time of Darwin’s CPU implementation and GPU accelerated Darwin, on the Intel machine. The CPU implementation is running with 8 threads

Figure 8 show the comparison of total execution times on the Intel machine, again with a logarithmic scale on the *y*-axis. The CPU implementation is running with 8 threads, which gives nearly the fastest execution time. The non-coalesced GPU implementation achieves a speedup of 11.8x over the CPU. With coalesced memory accesses the speedup becomes 109x. Figures 7 and 8 indicate that coalescing helps to improve the speedup by around 10x. This happens due to efficient utilization of large GPU global memory bandwidth.

The above results were obtained with the linear-gap penalty model which is also the default setting in Darwin. However, Darwin and hence our GPU acceleration also support the affine gap model. Table 1 shows the total execution time of the CPU and GPU implementation of Darwin for various values of *match*, *mismatch*, *gapo* and *gape* on the IBM machine. The CPU implementation is running with 64 threads. The table shows that the speedup nearly remains constant (20x-25x) regardless of the scoring scheme. Hence our GPU acceleration is equally effective for both linear and affine gap penalty scoring models.

**Table 1:** Runtimes and speedup for different scoring schemes on the IBM machine

	(2,-1,-2,-2)	(1,-3,-1,-1)	(5,-4,-10,-1)
CPU	31m15	21m28s	31m27s
GPU-coalesced	76.0	59.3	78.0
speedup	24.7	21.7	24.2

## Conclusions

Read overlapping is an important step in OLC based de novo assemblers. Darwin is a fast and accurate read overlapper for assembly of long DNA reads. It is based on the seed-and-extend paradigm. It has two stages: 1) D-SOFT, to compute the seeds and 2) GACT, to extend the seed hits on both sides to compute the overlap between two reads. The ASIC implementation of Darwin is shown to be hundreds of times faster than software based read overlappers. GPUs are cost-effective and easily accessible processing units that are used to accelerate many high performance applications. In this paper, we have shown a GPU implementation of Darwin which accelerates the Smith-Waterman alignment with traceback computation used in the GACT stage. We pack the sequences on the GPU and compute the Smith-Waterman alignment matrix by dividing the matrix into 8x8 submatrices. This helps to reduce the GPU memory accesses. To further reduce the memory transactions, writing to the traceback matrix is coalesced. We tested our implementation against the hand-optimized CPU implementation of Darwin. The results show that using the real Pacbio dataset, our GPU implementation is 24x faster than 64 IBM Power8 threads and 109x faster than 8 Intel Xeon threads, regardless of the scoring scheme (linear or affine gap). The GPU implementation can also be used to accelerate generic Smith-Waterman alignment of long DNA sequences. The implementation is available at https://github.com/Tongdongq/darwin-gpu.

## Availability and requirements

**Project name:** darwin-gpu**Project home page:**https://github.com/Tongdongq/darwin-gpu**Operating system(s):** Linux**Programming language:** C++, CUDA**Other requirements:** CUDA toolkit version 8 or higher.**License:** Apache 2.0**Any restrictions to use by non-academics:** Not applicable

## Data Availability

Not applicable.

## Abbreviations

ASIC: Application Specific Integrated Circuit
CPU: Central Processing Unit
CUDA: Compute Unified Device Architecture
DNA: Deoxyribonucleic Acid D-SOFT: Diagonal-band Seed Overlapping based Filtration Technique
GACT: Genome Alignment using Constant memory Traceback
GASAL: GPU Accelerated Sequence Alignment Library
GPU: Graphical Processing Unit
NGS: Next Generation Sequencing
SM: Streaming Multiprocessor
SP: Streaming Processor

## References

[1] Kececioglu JD, Myers EW (1995). Combinatorial algorithms for dna sequence assembly. Algorithmica.

[2] Myers G, Tischler G, Cunial F, Pippel M. DAZZLER: Dresden Azzembler for Long Read DNA Projects. https://https://dazzlerblog.wordpress.com. Accessed 2 July 2019.

[3] Simpson JT, Durbin R (2012). Efficient de novo assembly of large genomes using compressed data structures. Genome Res.

[4] Pevzner PA, Tang H, Waterman MS (2001). An eulerian path approach to dna fragment assembly. Proc Natl Acad Sci U S A.

[5] Zerbino D, Birney E (2008). Velvet: algorithms for de novo short read assembly using de bruijn graphs. Genome Res.

[6] Simpson JT, Wong K, Jackman SD, Schein JE (2009). Abyss: a parallel assembler for short read sequence data. Genome Res.

[7] Luo R, Liu B, Xie Y, Li Z (2012). Soapdenovo2: an empirically improved memory-efficient short-read de novo assembler. Gigascience.

[8] Yatish Turakhia GB, Dally WJ (2018). Darwin: genomics co-processor provides up to 15,000X acceleration on long read assembly. Proceedings of the Twenty-Third International Conference on Architectural Support for Programming Languages and Operating Systems. ASPLOS ’18.

[9] Ahmed N, Lévy J, Ren S, Mushtaq H, Bertels K, Al-Ars Z (2019). GASAL2: a GPU accelerated sequence alignment library for high-throughput NGS data. BMC Bioinformatics.

[10] Ren S, Ahmed N, Bertels K, Al-Ars Z (2019). GPU accelerated sequence alignment with traceback for GATK HaplotypeCaller. BMC Genomics.

[11] Houtgast EJ, Sima V-M, Bertels K, Al-Ars Z (2018). Hardware acceleration of bwa-mem genomic short read mapping for longer read lengths. Comput Biol Chem.

[12] Smith TF, Waterman MS (1981). Identification of common molecular subsequences. J Mol Biol.

[13] Ahmed N, Bertels K, Al-Ars Z (2016). A comparison of seed-and-extend techniques in modern dna read alignment algorithms. 2016 IEEE International Conference on Bioinformatics and Biomedicine (BIBM).

[14] Altschul SF, Gish W, Miller W, Myers EW, Lipman DJ (1990). Basic local alignment search tool. J Mol Biol.

[15] Roberts M, Hayes W, Hunt BR, Mount SM (2004). Reducing storage requirements for biological sequence comparison. Bioinformatics.

[16] Rucci E, Garcia C, Botella G, De Giusti A, Naiouf M, Prieto-Matias M (2018). SWIFOLD: Smith-Waterman implementation on FPGA with OpenCL for long DNA sequences. BMC Syst Biol.

[17] Farrar M (2007). Striped smith–waterman speeds database searches six times over other SIMD implementations. Bioinformatics.

[18] Hirschberg DS (1975). A Linear Space Algorithm for Computing Maximal Common Subsequences. Commun ACM.

[19] Chao KM, Pearson WR, Miller W (1992). Aligning two sequences within a specified diagonal band. Comput Appl Biosci CABIOS.

[20] Trapnell C, Schatz MC (2009). Optimizing data intensive gpgpu computations for dna sequence alignment. Parallel Comput.

[21] de O Sandes EF, de Melo ACMA. Smith-waterman alignment of huge sequences with gpu in linear space. In: 2011 IEEE International Parallel Distributed Processing Symposium. Piscataway: IEEE: 2011. p. 1199–211. 10.1109/IPDPS.2011.114. https://ieeexplore.ieee.org/document/6012857/.

[22] Liu Y, Schmidt B (2014). CUSHAW2-GPU: Empowering Faster Gapped Short-Read Alignment Using GPU Computing. Des Test IEEE.

[23] Houtgast EJ, Sima VM, Bertels KLM, Al-Ars Z (2016). An efficient gpu-accelerated implementation of genomic short read mapping with bwa-mem. Proc. International Symposium on Highly-Efficient Accelerators and Reconfigurable Technologies.

[24] Hasan L, Kentie MA, Al-Ars Z (2011). Dopa: Gpu-based protein alignment using database and memory access optimizations. BMC Res Notes.

[25] Ahmed N, Mushtaq H, Bertels KLM, Al-Ars Z (2017). Gpu accelerated api for alignment of genomics sequencing data. Proc. IEEE International Conference on Bioinformatics and Biomedicine.

[26] Turakhia Y. Darwin: A co-processor for long read alignment. https://github.com/yatisht/darwin. Accessed 5 Nov 2018.

[27] Data release: 54x long-read coverage for PacBio-only de novo human genome assembly. 2014. https://www.pacb.com/blog/data-release-54x-long-read-coverage-for/. Accessed 2 July 2019.

